# Frozen Section Analysis and Real-Time Magnetic Resonance Imaging of Surgical Specimen Oriented on 3D Printed Tongue Model to Assess Surgical Margins in Oral Tongue Carcinoma: Preliminary Results

**DOI:** 10.3389/fonc.2021.735002

**Published:** 2021-12-09

**Authors:** Caterina Giannitto, Giuseppe Mercante, Luca Disconzi, Riccardo Boroni, Elena Casiraghi, Federica Canzano, Michele Cerasuolo, Francesca Gaino, Armando De Virgilio, Barbara Fiamengo, Fabio Ferreli, Andrea Alessandro Esposito, Paolo Oliva, Flavio Ronzoni, Luigi Terracciano, Giuseppe Spriano, Luca Balzarini

**Affiliations:** ^1^ Department of Diagnostic Radiology, Humanitas Clinical and Research Center Istituti di Ricovero e Cura a Carattere Scientifico (IRCCS), Milan, Italy; ^2^ Otorhinolaryngology Unit, Humanitas Clinical and Research Centre Istituti di Ricovero e Cura a Carattere Scientifico (IRCCS), Milan, Italy; ^3^ Department of Biomedical Sciences, Humanitas University, Milan, Italy; ^4^ Department of Supply Chain, Humanitas Clinical and Research Center Istituti di Ricovero e Cura a Carattere Scientifico (IRCCS), Rozzano, Milan, Italy; ^5^ Department of Computer Science (DI), Università degli Studi di Milano, Milan, Italy; ^6^ Department of Pathology, Humanitas Clinical and Research Center Istituti di Ricovero e Cura a Carattere Scientifico (IRCCS), Milan, Italy; ^7^ Department of Radiology, Azienda Socio Sanitaria Territoriale di Bergamo Ovest, Treviglio, Italy

**Keywords:** head and neck, virtual surgical planning, 3D printing, tumor, resection, surgical margins, *ex-vivo*

## Abstract

**Background:**

A surgical margin is the apparently healthy tissue around a tumor which has been removed. In oral cavity carcinoma, a negative margin is considered ≥ 5 mm, a close margin between 1 and 5 mm, and a positive margin ≤ 1 mm. Currently, the intraoperative surgical margin status is based on the visual inspection and tissue palpation by the surgeon and intraoperative histopathological assessment of the resection margins by frozen section analysis (FSA). FSA technique is limited and susceptible to sampling errors. Definitive information on the deep resection margins requires postoperative histopathological analysis.

**Methods:**

We described a novel approach for the assessment of intraoperative surgical margins by examining a surgical specimen oriented through a 3D-printed specific patient tongue with real-time Magnetic Resonance Imaging (MRI). We reported the preliminary results of a case series of 10 patients, prospectively enrolled, with oral tongue carcinoma who underwent surgery between February 2020 and April 2021. Two radiologists with 5 and 10 years of experience, respectively, in Head and Neck radiology in consensus evaluated specimen MRI and measured the distance between the tumor and the specimen surface. We performed intraoperative bedside FSA. To compare the performance of bedside FSA and MRI in predicting definitive margin status we computed the weighted sensitivity (SE), specificity (SP), accuracy (ACC), area under the ROC curve (AUC), F1-score, Positive Predictive Value (PPV), and Negative Predictive Value (NPV). To express the concordance between FSA and *ex-vivo* MRI we reported the jaccard index.

**Results:**

Intraoperative bedside FSA showed SE of 90%, SP of 100%, F1 of 95%, ACC of 0.9%, PPV of 100%, NPV (not a number), and jaccard of 90%, and *ex-vivo* MRI showed SE of 100%, SP of 100%, F1 of 100%, ACC of 100%, PPV of 100%, NPV of 100%, and jaccard of 100%. These results needed to be validated in a larger sample size of 21- 44 patients.

**Conclusion:**

The presented method allows a more accurate evaluation of surgical margin status, and the first clinical experiences underline the high potential of integrating FSA with *ex-vivo* MRI of the fresh surgical specimen.

## Introduction

A surgical margin is the apparently healthy tissue around a tumor that has been surgically removed. Most commonly, in oral cavity carcinoma, a margin larger than or equal to 5 mm is considered as “negative”, a margin between 1 and 5 mm as “close”, and a margin less than 1 mm as “positive” (1, 2). Radicality and negative margin status represent the most successful outcome in oral cancer surgery. Close or positive margins require re-resection or adjuvant (chemo)radiotherapy contributing to costs, morbidity, and reduced quality of life of the patients who have to undergo these treatments. Mitchell et al. (3) showed that in oral carcinoma five-year survival was 81%, 75%, and 54% for clear, close, and involved margins, respectively, which highlights the importance of clear margins.

Currently, the intraoperative surgical margin status is based on the visual inspection and tissue palpation by the surgeon during surgery and intraoperative histopathological assessment of the resection margins by frozen section analysis (FSA). FSA technique is limited and susceptible to sampling errors (4, 5). Definitive information on the deep resection margins requires postoperative histopathological analysis.

The margin revision of initially positive margins to ‘‘clear’’ based on FSA guidance does not equate to an initially negative margin and does not significantly improve local control. Prospective studies should determine what system of resected specimen analysis best predicts completeness of resection (6).

Real-time Magnetic Resonance Imaging (MRI) on surgical specimen has been used in a few previous studies (7, 8). In particular, Heidkamp et al. (8) showed a positive predictive value (PPV) and negative predictive value (NPV) for Oral Squamous Cell Carcinoma (OSCC) localization of 87-96% and 75-79%, respectively, and a PPV and NPV for identification of margins <5 mm of 5-38% and 87-91%.

When studying correlations between imaging and histological data, the different spatial resolution of the two methods can increase bias, and the evaluation of the piece of the organ as opposed to the whole organ without orientation references could be challenging for the pathologist. The introduction of a 3D-printed anatomic model of the tongue of the patient, obtained from staging MRI for surgical specimen orientation, reproducing the anatomic context from which the specimen has been excised could improve surgeon, pathologist, and radiologist communication in the assessment of margins.

The employment of MRI to examine the surgical specimen oriented through the 3D model could allow for a better macroscopic radial margin evaluation and measurement of the distance to all margins, avoiding sampling errors of FSA.

The purpose of this paper was to report the preliminary results of the diagnostic accuracy of FSA and MRI in evaluating intraoperative surgical margins in oral tongue carcinoma, by examining the surgical specimen oriented through the 3D-printed specific patient tongue model.

## Materials and Equipment

**Figure 1:**
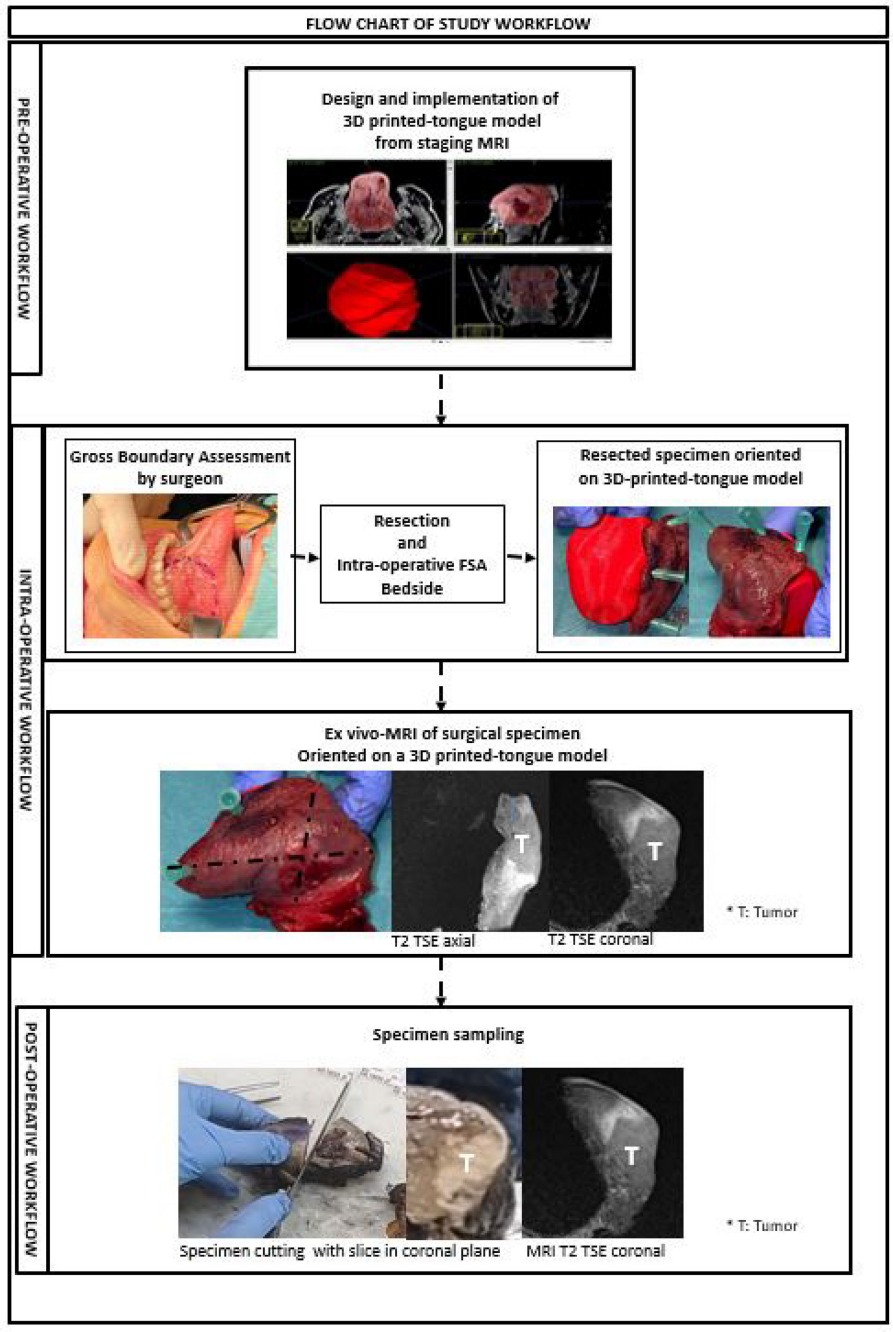
This figure shows pre-operative, intra-operative and post-operative steps of the study workflow.

We described the steps of the process in a sequential workflow, diagrammed in a flow chart (**Figure 1**), to clarify how these steps have been executed.

### Pre-Operative Workflow: Design and Implementation of 3D Printed-Tongue Model

**Figure 2:**
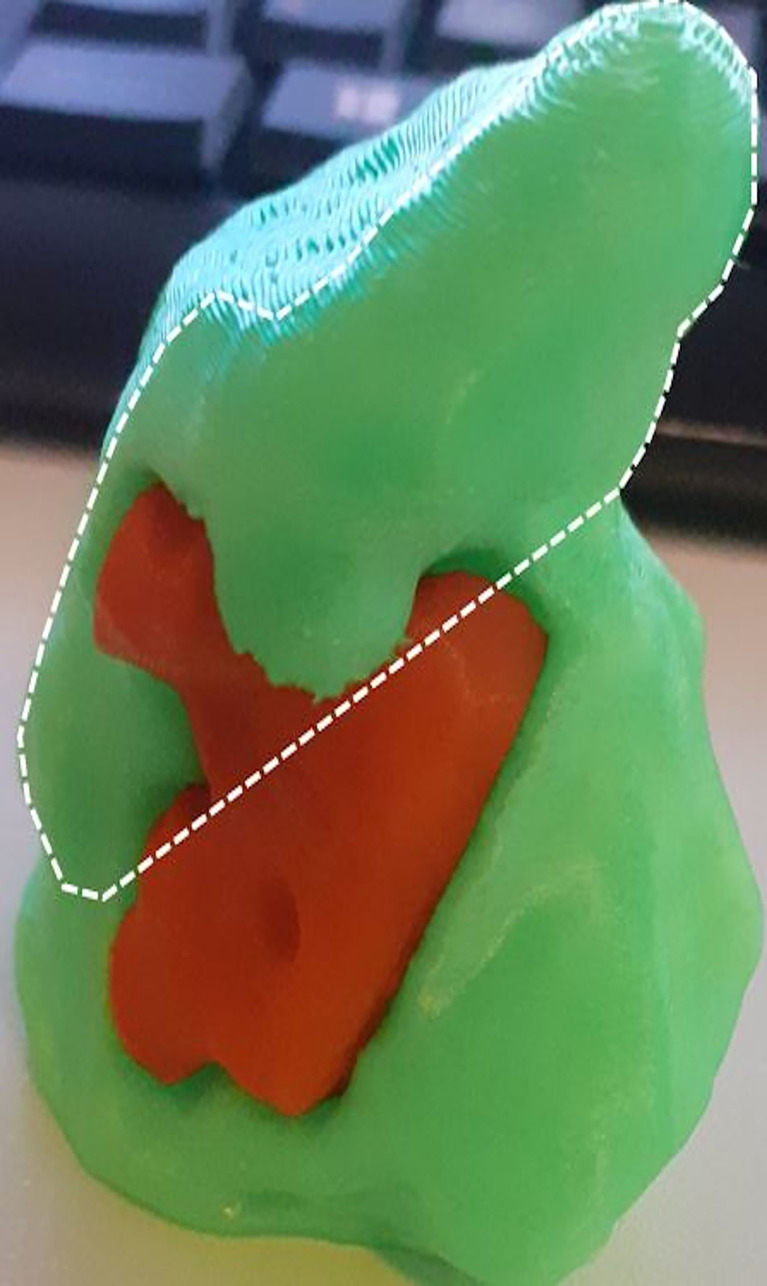
Lateral view of the 3D-printed model shows the protuted tongue (inside the dotted line), the floor of the mouth (outside the dotted line), and the tumor in red.

We obtained a 3D-printed model of the patient’s tongue on which the area which should have been resected has been reproduced. We implemented the model by the 3D post-contrast fat suppressed gradient-echo T1weighted sequence (VIBE) of staging MRI examinations on a 1.5T MRI System (Magnetom Aera, Siemens Healthineers, Erlangen, Germany) using a phased array surface coil. 3D post-contrast anonymized images demonstrating a good contrast between the tumor and the tongue parenchyma were selected and transferred into ITK-SNAP, a software application used to segment structures in 3D medical images (http://www.itksnap.org/pmwiki/pmwiki.php). The tongue tumor and tongue parenchyma were segmented as separate anatomical regions of interest (ROIs). For all ROIs, both threshold and manual editing were performed to ensure that only the anatomy of interest would be selected. Each ROI was converted to a separate 3D object and combined into a 3D virtual model. The segmentation data, in DICOM format, were converted to STL format so that the 3D printer could recognize them. Surgeons and radiologists segmented tongue tumor, and a 3D virtual model (**Figure 2**) of the tongue with the pathological area was printed by a 3D printer (VERVE, Kentstrapper, Florence Italy).

### Intraoperative Workflow: Intraoperative FSA and *Ex Vivo*-MRI of Surgical Specimen Oriented on a 3D Printed-Tongue Model

**Figure 3:**
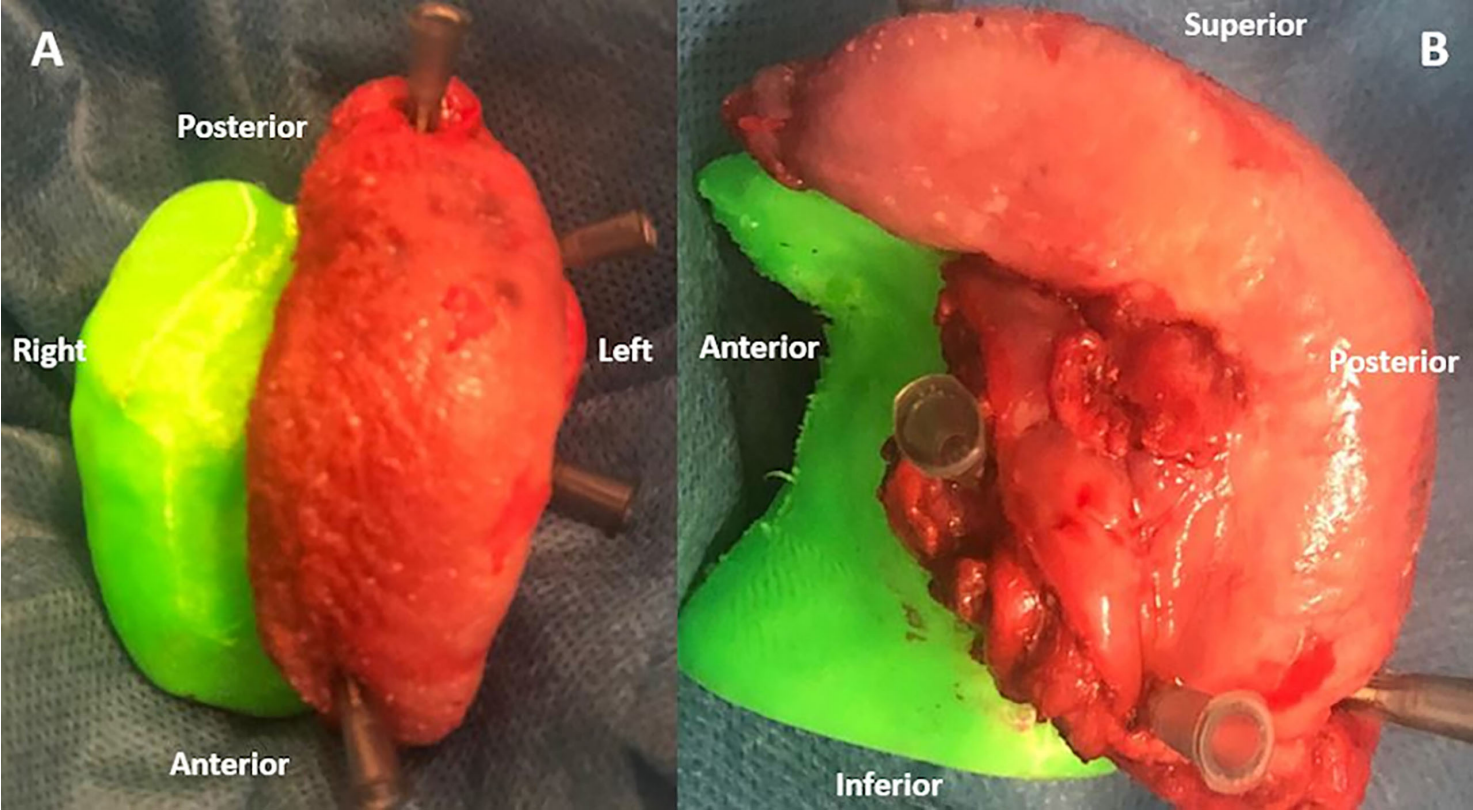
Superior **(A)** and Lateral **(B)** views of surgical specimen of a left hemiglossectomy oriented on a 3D-printed model of the protuted tongue and the floor of the mouth obtained from the staging MRI of the patient.

**Figure 4:**
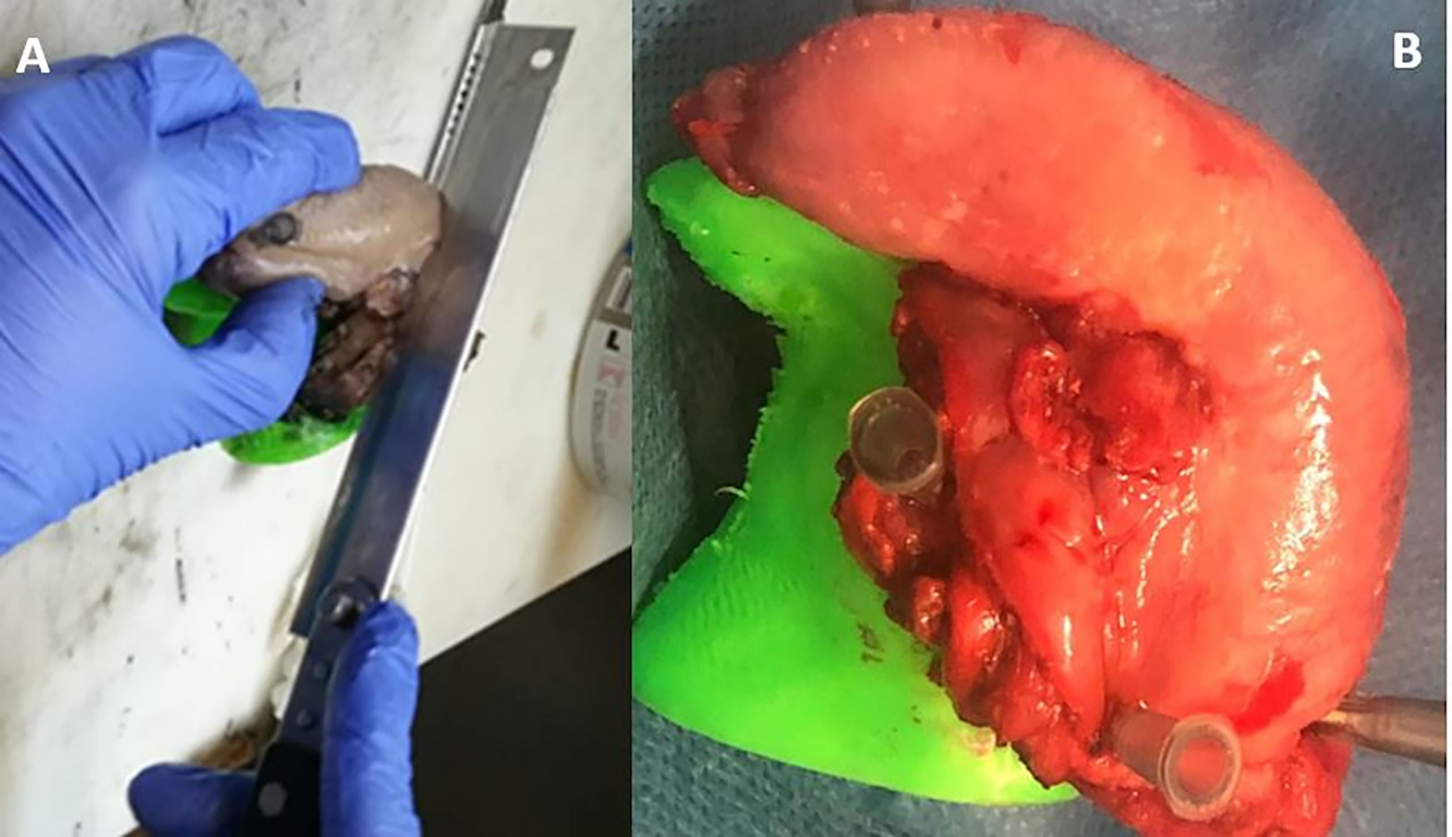
Dissection of the specimen into 3–5 mm slices **(A, B)** parallel to MRI planes to evaluate the distance from the tumour to the surface of the specimen.

The surgeon determined the intended boundaries of resection and, following resection, sampled the tumor bed to establish intraoperative margin status. The positive margins at FSA have been radicalized. While maintained at the operating room, fresh specimens were fixed on the 3D-printed model for the correct orientation of the resected surgical specimen (**Figures 3**, **4**). The specimen was immersed in perfluoropolyether (Galden, Solvay Solexis, Thorofare, New Jersey) to eliminate magnetic susceptibility artifacts arising from the air tissue transition (7).The specimen oriented on the 3D model was placed on an MRI phantom with a reference placed on the tumor. A four channel phased array surface carotid coil (Magnetom Aera, Siemens, Erlangen, Germany) was mounted underneath and on top of the 3D model which was positioned in a 1.5 T clinical MRI system (Magnetom Aera, Siemens, Erlangen, Germany). Axial, Coronal, Sagittal T2‐weighted (T2W) turbo spin echo (TSE) sequences (using Field of View (FoV) read 130 mm, FoV phase 100.0%, slice thickness 3.0 mm, TR 3430.0 ms, TE 92.0 ms, Averages 3) and Diffusion weighted spin‐echo echo planar images (using FoV read 140 mm, FoV phase 100.0%, slice thickness 3.0 mm, TR 3500 ms,TE 55.0 ms) were acquired. Apparent diffusion coefficient (ADC) maps were calculated based on acquired b values of 50, 500, and1000  s/mm2 using the standard post processing available on the MRI system.

### Post-Operative Workflow: Specimen Sampling

Following MRI acquisition, surgical specimen oriented on the 3D-printed tongue model was transported to the pathology laboratory for formalin fixation. Next, the specimen oriented on the 3D model was completely cut up into 4 mm thick slices parallel to the coronal plane of MRI evaluation (**Figure 4**) and whole-mount paraffin was embedded to evaluate the macroscopic depth of invasion of the tumor and the radial distance of the tumor to all of the margins. In addition to this, the radial margin at the periphery has been submitted. The serial slices of the specimen were sequentially laid out, numbered, and photographed.

## Methods

### Study Design and Patients

This was a pilot observational prospective mono-institutional cohort study including all consecutive adult patients with a histological diagnosis of OSCC who underwent a primary surgical treatment of lingual resection (hemiglossectomy or partial glossectomy) between February 2020 and April 2021. We prospectively included all the cases with an intraoperative margin status evaluation by FSA and *ex-vivo* MRI. Institutional review board approval and informed consent for all the patients enrolled were obtained.

### Qualitative and Quantitative Analysis of *Ex-Vivo* MRI

The mean time duration of *Ex-vivo* MRI examination was 22.7 minutes (range 16-40 minutes).

For the qualitative analysis, two radiologists (CG and LB, dedicated head and neck radiologists with respectively 5 and more than 20 years experience), in consensus, evaluated the image quality of the acquired MRI series, enabling visualization of even small structures.

**Figure 5:**
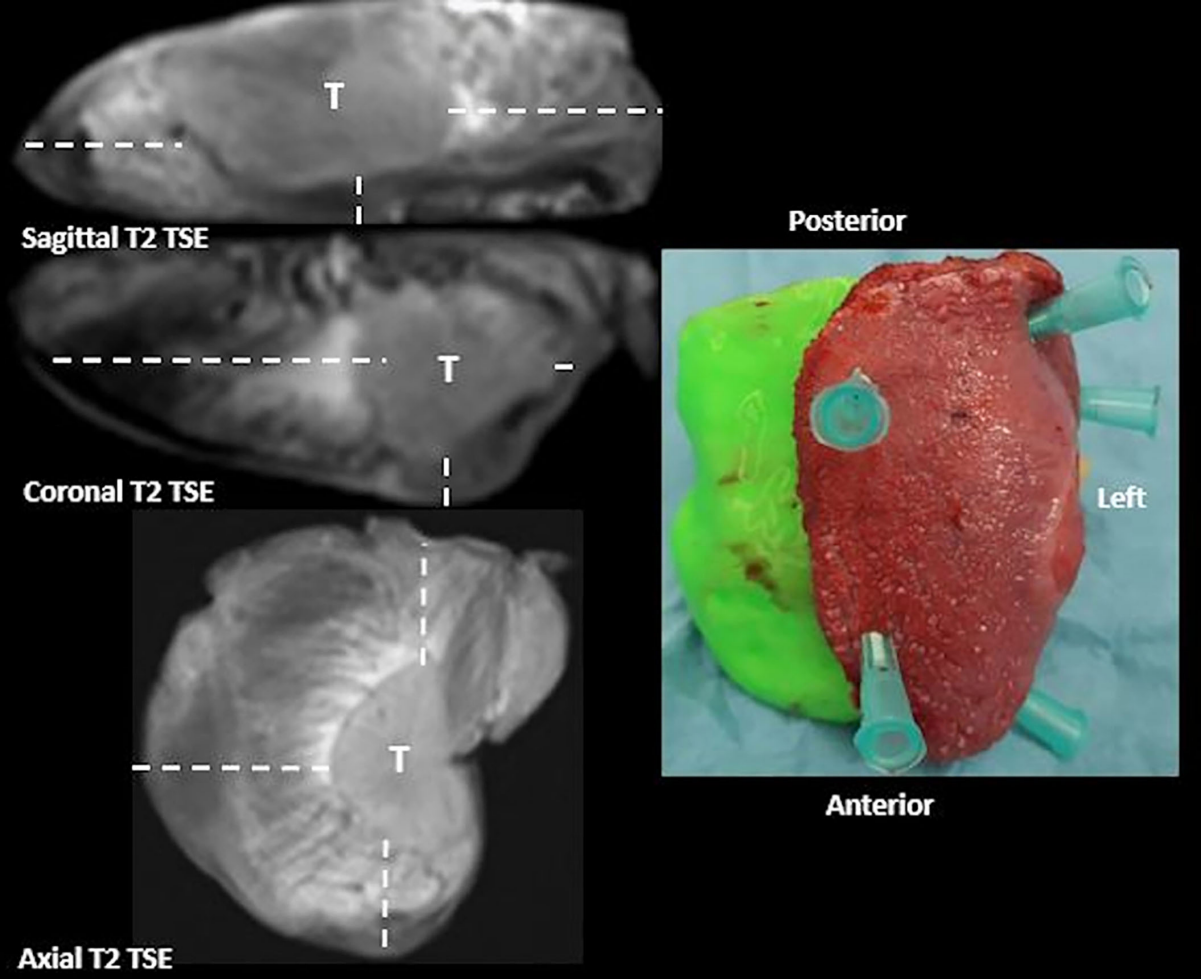
Coronal, sagittal, and axial T2 TSE MRI sequences of the surgical specimen oriented on a 3D-printed model. Multiplanar MRI sequences show the tumor (T) and its macroscopic radial distance (dotted line) from the surface of the specimen in all the planes.

They, blinded to histological results, radially measured the distance (in millimeters) from all the margins to the tumor (**Figure 5**).

To differentiate tumor from edema they calculated ADC values within ROI drawn on the tumor and surrounding tissue.

They considered a margin clear if > 5mm, close if =1–5 mm, and involved if *<*1 mm.

The patients were stratified into three groups depending on the analysis of their margins. The three groups were negative, close, and involved.

### Reference Standard: Radio-Pathological Correlation

A dedicated head and neck pathologist with more than of ten years of experience (BF) without knowledge of the MRI results annotated tumor location and measured the distance from the tumor to the margins, which was considered the gold standard.

The serial coronal slices were correlated with the T2 coronal sequences of *ex-vivo* MRI of specimen oriented on the 3D-printed tongue model (**Figures 1**, **4**).

The patients were stratified into three groups depending on the final analysis of their margins on the specimen. The three groups were negative, close, and involved.

### Statistical Analysis

#### Diagnostic Accuracy

To compare the performance of the initial bedside FSA and *ex-vivo* MRI in predicting definitive negative, close, and involved margins we computed the weighted sensitivity(SE), specificity(SP), accuracy(ACC), area under the ROC curve (AUC), F1-score, Positive Predictive Value (PPV), and Negative Predictive Value (NPV), where the weights are proportional to the cardinality of each class.

In addition to this, to express the concordance between FSA and *ex-vivo* MRI, we reported the jaccard index, computed as the number of equal classifications, divided by the number of total samples. Final pathological diagnosis was the gold standard.

#### Intended Sample Size for Conclusive Results

In a study published in the literature (6), 640 consecutive patients over an 11-year period with at least five years’ follow up were studied. A total of 213 patients (33%) had resection margins that were clear (5 mm or more), 314 (49%) were close (1 - 4.9 mm), and 113 (18%) were involved (0 - 0.9 mm). The required sample size was determined in order to detect a significant difference with an accuracy 0.2-0.3 times greater than 0.50 (which practically corresponds to a random classifier) with a power of 0.90. Assuming equal variance, a total of 21-44 patients should be enrolled to report conclusive results.

## Results

### Participants

We enrolled 10 patients (6 females and 4 males with a median age 53.1 years, range 30-89) with a histological diagnosis of squamous cell carcinoma of the oral cavity (OSCC) who underwent surgery (5 hemiglossectomies and 5 partial glossectomies) for oral tongue squamous carcinoma between February and April 2021. The definitive margin status was negative in nine cases and positive in one case. In *ex-vivo* MRI, margin status was negative in nine cases and positive in one case (revision was not performed in this case because FSA was negative). The final pathological T status was T1 in one case, T2 in seven cases, and T3 in two cases. In the 10 cases, the mean maximum diameter of the tumor at the final histological diagnosis was 22.3 (range 10-37 mm). The average time taken for each MRI examination was 24.7 minutes (range 16-38 minutes). The T2 series of MRI was therefore the series that was matched to histology and was subsequently annotated.

### Preliminary Results

MRI succeeded in margin prediction in all the cases, and FSA failed in one case.

In this group of patients initial bedside FSA showed SE of 90%, SP of 100%, F1 of 95%, ACC of 90%, PPV of 100%, NPV (not a number), and jaccard of 90%, and *ex-vivo* MRI showed SE of 100%, SP of 100%, F1 of 100%, ACC of 100%, PPV of 100%, NPV of 100%, and jaccard of 100%.

These results needed to be validated in a larger sample size of 21- 44 patients.

## Discussion

Intraoperative assessment of the resection margins can provide valuable information, enabling additive resection to obtain negative margin status. Despite this, the clinical value and the method of assessing the intraoperative margin are not well defined (5, 9, 10). Many factors influence the evaluation of surgical margins, making it more or less adequate; these include the sampling of the margins (from the block sample compared to the surgical defect alone), the ability and methods used to determine the distance to margins, the communication between the surgeon and the pathologist involving the specimen, orientation and areas of concern, and the subsite in the head and neck (11).

Several techniques aiming for intraoperative assessment of surgical margins in oral cavity/tongue squamous cell carcinoma have been investigated. These include elastic scattering spectroscopy (12) fluorescence (13–15), hyperspectral imaging (16), optical coherence tomography (17), spectroscopy (18), ultrasound (19, 20) and intraoperative slicing of the whole specimen by the pathologist (21). Only MRI, ultrasound, and intraoperative slicing of the whole specimen by the pathologist can allow sampling of the entire specimen and/or probing depth of the lesion.

With our study, we have found it helpful to report and demonstrate to our surgical team the gross distance to all margins by using the intraoperative *ex- vivo* MRI, and to improve surgeon and pathologist communication by introducing a 3D-printed tongue model to allow pathologists to understand specimen orientation and to learn what margins will be revised based on gross impression.

To our knowledge this is the first study in which a surgeon provided orientation of the specimen on the 3D-printed model of the tongue with the reproduced bed of resection. Generally, the surgeon provides orientation by designating one or two points on the specimen.

The specimen orientation on the 3D-printed model from patient MRI facilitates review and correlation.

By maintaining the orientation of the specimen, the pathologist is facilitated in noting the distance of the tumor from each margin and in communicating the site of positive and close margins to the submitting surgeon.

While most surgeons sample margins only from the surgical bed without margin assessment from the resection specimen, as demonstrated by a survey of American Head and Neck Society members (22), we introduced *ex-vivo* MRI to outcome the limit of the lack of a true measurement of the distance of the invasive tumor from the resection margin. According to FSA, a margin can only be determined as positive or negative and the margin presented separately can be thin <5 mm in thickness. Additionally, without a defined margin orientation it is not clear how the separately submitted margins reflect the true areas of the en block specimen that are noted to be close or positive. In our preliminary experience, *ex vivo* MRI was more accurate than intraoperative FSA in predicting margin status.

The results of a previous study (7) showed high specificity and low sensitivity of MRI in identifying margins less than 5 mm. According to these results, *ex-vivo* MRI assessment of the pathologic en bloc specimen could direct intra-operative defect-derived FSA margin assessment and margin revision, determining the closest margins on gross assessment and requiring separately submitted tissues of correct size to have negative margins. The orientation of the specimen on the 3D model could allow a better match between the separately submitted margins and the true areas of the en bloc specimen that are noted to be close or positive. This could be because the revised margin is not taken from the correct location (23).

Some limitations of our study should be discussed. First, the sample size was small and heterogeneous. Our cohort contained a broad range of cases with various T classifications. Furthermore, a relatively small proportion of the margins were less than 5 mm. As established in our statistical analysis on intended sample size, our preliminary results should be validated in a larger cohort of patients.

The limitation of *ex- vivo* MRI could be the lack of identification of microscopic tissue changes. Conventional MRI could not eneable thin differentiations between tumor and surrounding tissue in the presence of edema. Diffusion weighted imaging (DWI) can be used to evaluate the rate of microscopic water diffusion within tissues. DWI may be measured by means of apparent diffusion coefficient (ADC). Areas of decreased ADC values within tumors could be used as a powerful imaging biomarker of cancer (24, 25).

Tumor shrinkage after formalin fixation could lead to an underestimation of tumor margins (26). This could be demonstrated by performing *ex-vivo* MRI of the specimen after formalin fixation. We performed MRI after formalin fixation in one case but the changes of the signal intensity of the specimen did not allow us to evaluate the effect of shrinkage.

Other limitations were the inexperience of the MRI readers, who had experience with *in vivo* applications of MRI but not in *ex-vivo* MRI of tongue resection specimens, and the fact that only one pathologist evaluated histopathology.

*Ex-vivo* MRI is a costly and time consuming process. It requires the use of MRI equipment, subtracting time for performing diagnostic tests. It also requires excellent communication and coordination between surgeons, operating room staff, pathologists, and radiologists to avoid additional operating time. Considering that the operating times for these types of complex resections and reconstructions, with the maximum operating time of 160 minutes in which flap reconstruction was not required and 700 minutes of microvascular reconstruction, MRI does not unreasonably lengthen the time in the operating room (21). This intervention has the potential to both reduce rates of close pathological margins and the need for postoperative radiotherapy, as shown in our preliminary experience with the detection of positive margins not detected by intraoperative FSA.

In conclusion, considering the staffing and expensive nature of integrating FSA with *ex-vivo* MRI of the fresh specimen for evaluation on macroscopic proximity of the tumor to the margins of resection, this intervention can be performed with some planning and coordination between the radiologists, pathologists, and surgeons in a selected subset of patients most likely to benefit by avoiding adjuvant radiotherapy. Finally, we would recommend consideration of intraoperative MRI tumor margin assessment for selected cases potentially be cured with surgery alone, i.e. T1/T2N0 tumor.

## Data Availability Statement

The raw data supporting the conclusions of this article will be made available by the authors by contacting the corresponding author.

## Ethics Statement

The study was reviewed and approved by the Humanitas Research Hospital, Rozzano. The patients/participants provided their written informed consent to participate in this study.

## Author Contributions

CG, LB, GS, and GM conceived of the presented idea. CG developed the theory and performed the computations. CG, GM, LB, and FF verified the analytical methods. GS encouraged CG to investigate margin status and supervised the findings of this work. All authors discussed the results and contributed to the final manuscript. CG, GM, LD, FC, MC, FG, RB, and BF carried out the experiment. CG wrote the manuscript with support from GM. RB and PO fabricated the 3D model sample. AD, FR, AE, FF, and LT helped supervise the project. CG and EC developed the theoretical formalism, performed the analytic calculations and performed the numerical simulations and contributed to the interpretation of the results. All authors contributed to the article and approved the submitted version.

## Conflict of Interest

The authors declare that the research was conducted in the absence of any commercial or financial relationships that could be construed as a potential conflict of interest.

## Publisher’s Note

All claims expressed in this article are solely those of the authors and do not necessarily represent those of their affiliated organizations, or those of the publisher, the editors and the reviewers. Any product that may be evaluated in this article, or claim that may be made by its manufacturer, is not guaranteed or endorsed by the publisher.
